# Cyclic AMP‐hydrolyzing phosphodiesterase inhibitors potentiate statin‐induced cancer cell death

**DOI:** 10.1002/1878-0261.12775

**Published:** 2020-08-25

**Authors:** Joseph Longo, Aleksandra A. Pandyra, Paweł Stachura, Mark D. Minden, Aaron D. Schimmer, Linda Z. Penn

**Affiliations:** ^1^ Princess Margaret Cancer Centre University Health Network Toronto Canada; ^2^ Department of Medical Biophysics University of Toronto Toronto Canada; ^3^ Department of Molecular Medicine II Medical Faculty Heinrich Heine University Düsseldorf Germany; ^4^ Department of Gastroenterology, Hepatology, and Infectious Diseases Heinrich Heine University Düsseldorf Germany

**Keywords:** cilostazol, dipyridamole, mevalonate pathway, phosphodiesterase inhibitor, SREBP2, statins

## Abstract

Dipyridamole, an antiplatelet drug, has been shown to synergize with statins to induce cancer cell‐specific apoptosis. However, given the polypharmacology of dipyridamole, the mechanism by which it potentiates statin‐induced apoptosis remains unclear. Here, we applied a pharmacological approach to identify the activity of dipyridamole specific to its synergistic anticancer interaction with statins. We evaluated compounds that phenocopy the individual activities of dipyridamole and assessed whether they could potentiate statin‐induced cell death. Notably, we identified that a phosphodiesterase (PDE) inhibitor, cilostazol, and other compounds that increase intracellular cyclic adenosine monophosphate (cAMP) levels potentiate statin‐induced apoptosis in acute myeloid leukemia and multiple myeloma cells. Additionally, we demonstrated that both dipyridamole and cilostazol further inhibit statin‐induced activation of sterol regulatory element‐binding protein 2, a known modulator of statin sensitivity, in a cAMP‐independent manner. Taken together, our data support that PDE inhibitors such as dipyridamole and cilostazol can potentiate statin‐induced apoptosis via a dual mechanism. Given that several PDE inhibitors are clinically approved for various indications, they are immediately available for testing in combination with statins for the treatment of hematological malignancies.

## Graphical abstract

Dipyridamole, an antiplatelet drug, synergizes with statins to induce tumor‐specific apoptosis. Here, we took a pharmacological approach to dissect the mechanism of action. By evaluating compounds that phenocopy the individual activities of dipyridamole, we uncovered that cAMP‐hydrolyzing phosphodiesterase inhibitors can potentiate statin‐induced apoptosis via a dual mechanism. This approach may be useful when interrogating novel functions of other repurposed drugs.

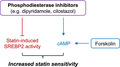

**Abbreviations**
AML: acute myeloid leukemia
ANOVA: analysis of variance
cAMP: cyclic adenosine monophosphate
cGMP: cyclic guanosine monophosphate
GGPP: geranylgeranyl pyrophosphate
HMG‐CoA: 3‐hydroxy‐3‐methylglutaryl coenzyme A
HMGCR: HMG‐CoA reductase
MM: multiple myeloma
MVA: mevalonate
PDE: phosphodiesterase
PKA: protein kinase A
qRT–PCR: quantitative reverse transcription–PCR
SD: standard deviation
sgRNAs: small guide RNAs
SREBP: sterol regulatory element‐binding protein

## 1 Introduction

The synthesis of cholesterol and other isoprenoids via the mevalonate (MVA) pathway is tightly regulated to maintain homeostasis. In many cancer cells, an increased dependency on isoprenoid biosynthesis for growth and survival confers sensitivity to the statin family of drugs, which inhibits the rate‐limiting enzyme of the MVA pathway, HMG‐CoA reductase (HMGCR) [1]. However, in normal cells and many cancer cells, treatment with statins activates the transcription factor sterol regulatory element‐binding protein 2 (SREBP2), which functions to upregulate genes involved in MVA metabolism to restore homeostasis. Activation of this feedback response has been associated with statin resistance in cancer cells [2, 3, 4]. In contrast, subsets of cancer cells that do not induce this feedback loop following statin treatment readily undergo apoptosis [2, 4].

**Fig. 1:**
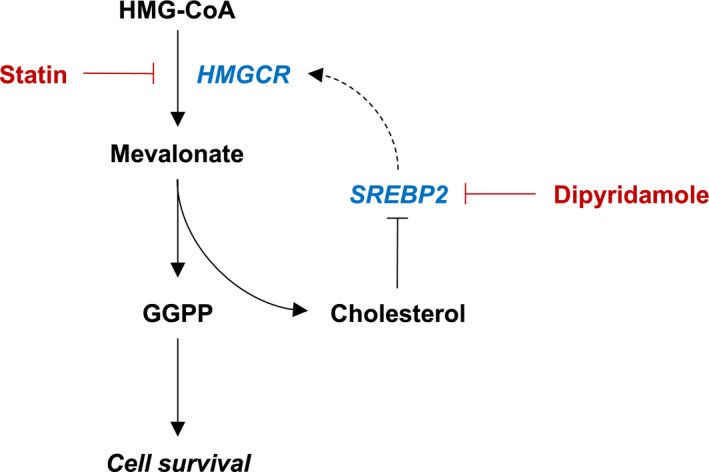
Dipyridamole inhibits the sterol‐regulated feedback loop of the MVA pathway. Schematic representation of the MVA pathway. Statins inhibit the rate‐limiting enzyme of the pathway, HMGCR, which catalyzes the conversion of HMG‐CoA to MVA. MVA is subsequently used to synthesize various metabolites that are important for cell growth and survival, including GGPP and cholesterol. Statin‐mediated cholesterol depletion induces the cleavage and activation of SREBP2, which in turn induces the transcription of genes involved in MVA metabolism to restore homeostasis. We previously identified that the drug dipyridamole can inhibit statin‐induced SREBP2 activation; however, the mechanism by which dipyridamole inhibits SREBP2 cleavage remains poorly understood.

We previously demonstrated that inhibition of this feedback response via RNAi‐mediated knockdown of SREBP2 potentiates statin‐induced cell death in lung and breast cancer cell lines [5]. Moreover, through a drug screening approach, our laboratory identified that the drug dipyridamole, an antiplatelet agent approved for secondary stroke prevention, can synergize with statins to induce apoptosis in acute myeloid leukemia (AML) and multiple myeloma (MM) cells [6]. We further demonstrated that dipyridamole inhibits statin‐induced SREBP2 cleavage and activation, thus abrogating the restorative feedback loop of the MVA pathway (Fig. 1) [6]. Since these initial observations in AML and MM, dipyridamole has been shown to inhibit statin‐induced SREBP2 activation and potentiate statin‐induced cell death in breast [3] and prostate [4] cancer; however, the mechanism by which dipyridamole inhibits SREBP2 and potentiates statin‐induced cancer cell death remains poorly characterized.

In this manuscript, we present data to suggest that the synergistic anticancer interaction between statins and dipyridamole is twofold. In part, the ability of dipyridamole to function as a phosphodiesterase (PDE) inhibitor and increase cyclic adenosine monophosphate (cAMP) levels sensitizes cancer cells to statin‐induced apoptosis. Additionally, dipyridamole and another cAMP‐hydrolyzing PDE inhibitor, cilostazol, are able to inhibit statin‐induced SREBP2 activity, and thus potentiate the proapoptotic activity of statins through a second, cAMP‐independent mechanism. Collectively, these data warrant further investigation into the combination of a statin and cAMP‐hydrolyzing PDE inhibitor for the treatment of hematological malignancies.

## 2 Materials and methods

### 2.1 Cell culture and compounds

KMS11, LP1, OCI‐AML‐2, and OCI‐AML‐3 cell lines were cultured as described previously [6]. S49 wild‐type (CCLZR352) and kin‐ (CCLZR347) cells were purchased from the University of California, San Francisco (UCSF) Cell Culture Facility and were cultured in Dulbecco's modified Eagle medium supplemented with 10% heat‐inactivated horse serum, 100 units·mL^−1^ penicillin, and 100 μg·mL^−1^ streptomycin. Cell lines were routinely confirmed to be mycoplasma‐free using the MycoAlert Mycoplasma Detection Kit (Lonza, Mississauga, Canada). Atorvastatin calcium (21CEC Pharmaceuticals Ltd., Markham, Canada) and fluvastatin sodium (US Biological, Burlington, Canada) were dissolved in ethanol. Dipyridamole (Sigma, Oakville, Canada), cilostazol (Tocris Bioscience, Burlington, Canada), *S*‐(4‐nitrobenzyl)‐6‐thioinosine (NBMPR) (Tocris Bioscience), 4‐{[3′,4′‐(methylenedioxy)benzyl]amino}‐6‐methoxyquinazoline (MBMQ) (Calbiochem, Oakville, Canada), fasentin (Sigma), and forskolin (Sigma) were dissolved in DMSO. Mevalonate and dibutyryl‐cAMP (db‐cAMP) were purchased from Sigma and dissolved in water. Geranylgeranyl pyrophosphate (GGPP) (methanol : ammonia solution) was purchased from Sigma.

### 2.2 Cell viability assays

3‐(4,5‐Dimethylthiazol‐2‐yl)‐2,5‐diphenyltetrazolium bromide (MTT) assays were performed as previously described [7]. Briefly, cells were seeded at 15 000–20 000 cells/well in 96‐well plates and treated as indicated for 48 h. Percent cell viability was calculated relative to cells treated with solvent control(s). Fluvastatin dose–response curves were plotted, and area under the dose–response curve (AUC) values were computed using graphpad prism v6 software (San Diego, CA, USA).

### 2.3 Cell death assays

Cells were seeded at 750 000 cells/well in 6‐well plates and treated as indicated for 48 h. For propidium iodide (PI) staining, cells were fixed in 70% ethanol for at least 24 h, stained with PI, and analyzed by flow cytometry for the % pre‐G1 DNA population as a measure of cell death, as previously described [2]. For Annexin V staining, cells were processed and stained using the Annexin V‐FITC Apoptosis Kit (BioVision Inc., Burlington, Canada) as per the manufacturer's protocol, or washed and stained as indicated in Annexin V Binding Buffer (BD Biosciences, Mississauga, Canada). Apoptosis assays using primary AML cells were performed as described previously [6]. Patient samples were obtained with informed consent under a protocol approved by the University Health Network Research Ethics Board in accordance with the Declaration of Helsinki.

### 2.4 CCLE data mining

RNA sequencing data for the selected AML and MM human cell lines from the Cancer Cell Line Encyclopedia (CCLE) [8] were analyzed using the UCSC Xena Functional Genomics Explorer (https://xenabrowser.net/) [9].

### 2.5 CRISPR/Cas9‐mediated gene knockout

Independent small guide RNAs (sgRNAs) that target *PRKACA* were cloned into lentiCRISPR v2 (Addgene plasmid #52961, Watertown, MA, USA). A sgRNA targeting a random locus on chromosome 10 was used as a negative control. HEK‐293Tv cells were co‐transfected with the sgRNA constructs, pMD2.G and psPAX2 using calcium‐phosphate. LP1 cells were transduced with the lentiviral supernatants in the presence of 8 μg·mL^−1^ polybrene, after which they were selected with 1 μg·mL^−1^ puromycin. The sequences for the sgRNAs were obtained from Ref. [10] and are as follows:

gC10 Random: AAACATGTATAACCCTGCGC
gPRKACA #1: ACGAATCAAGACCCTCGGCA
gPRKACA #2: AGATGTTCTCACACCTACGG

### 2.6 Immunoblotting

For proteins other than HMGCR, immunoblotting was performed as previously described [4], using the following primary antibodies: SREBP2 (1 : 250; BD Biosciences, 557037), Actin (1 : 3000; Sigma, A2066), PKA C‐α (1 : 1000; Cell Signaling Technology, #4782), α‐Tubulin (1 : 3000; Calbiochem, CP06), and Ku80 (1 : 3000; Cell Signaling Technology, #2180). For HMGCR immunoblots, cells were seeded at 750 000 cells/well in 6‐well plates and treated as indicated for 24 h. Whole cell lysates were prepared by washing cells twice with cold PBS and lysing cells in ~ 80 μL of buffer (20 mm Tris pH 7.5, 150 mm NaCl, 1 mm EDTA, 1 mm EGTA, 0.5% Triton X‐100, protease inhibitors) on ice for 30 min. Lysates were cleared by centrifugation and protein concentrations determined using the Pierce 660 nm Protein Assay Kit (Thermo Fisher Scientific). Dithiothreitol (DTT) was added to a final concentration of 1 m. 4x Laemmli sample buffer was then added to the DTT‐containing lysates at room temperature. Samples were not boiled to limit aggregation of membrane proteins. Blots were probed with primary antibodies against HMGCR (A9) (1 : 1000; prepared in‐house) and actin.

### 2.7 Quantitative RT–PCR

Total RNA was isolated using TRIzol Reagent (Invitrogen, Mississauga, Canada). cDNA was synthesized from 500 ng RNA using SuperScript III (Invitrogen), or RNA was directly used for RT–PCR analysis using the iTaq Universal Probe One‐Step Kit (Bio‐Rad, Mississauga, Canada), according to the manufacturer's instructions. Quantitative reverse transcription–PCR (qRT–PCR) was performed using TaqMan probes (Applied Biosystems, Mississauga, Canada) for the following genes: *HMGCR* (Hs00168352), *HMGCS1* (Hs00266810), *INSIG1* (Hs01650979), and *GAPDH* (Hs99999905).

### 2.8 Intracellular cAMP quantification

Intracellular levels of cAMP were measured using the Cyclic AMP Chemiluminescent Immunoassay Kit (Cell Technology, Hayward, CA, USA) as per the manufacturer's protocol. Briefly, 1.5 × 10^6^ cells/well (6‐well plate) were incubated with the compounds as indicated, washed with PBS, and lysed in 150 µL of the provided lysis buffer.

## 3 Results

### 3.1 The cAMP‐hydrolyzing PDE3 inhibitor cilostazol phenocopies dipyridamole to potentiate statin‐induced cancer cell death

**Fig. 2:**
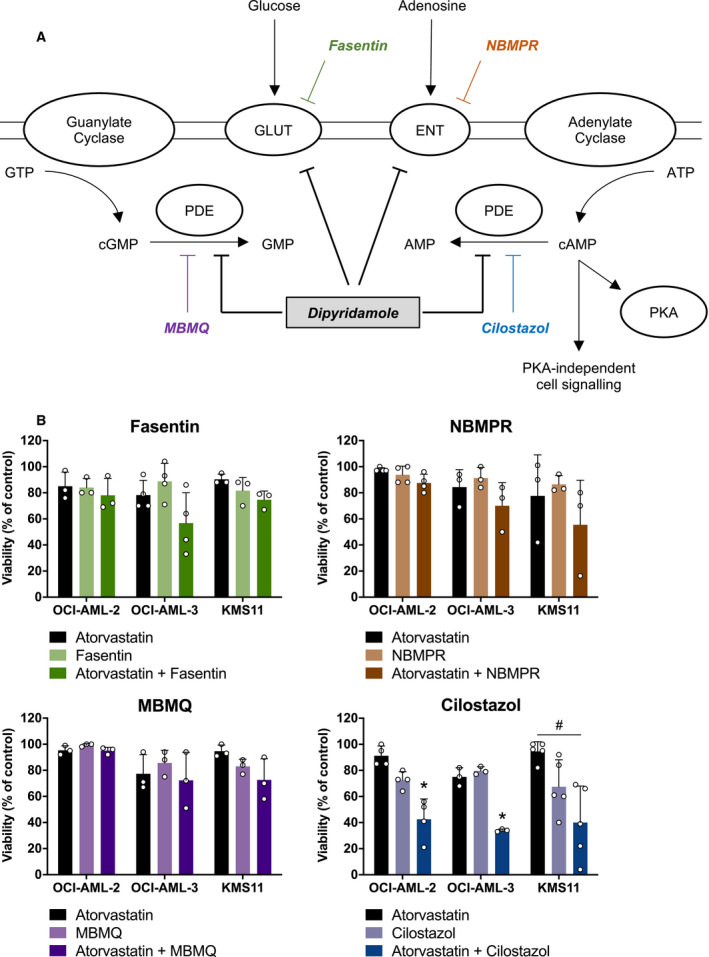
The cAMP‐hydrolyzing PDE3 inhibitor cilostazol phenocopies dipyridamole to potentiate statin‐induced cancer cell death. (A) Schematic representation of the reported targets of dipyridamole and additional compounds that target these proteins. ENT, equilibrative nucleoside transporter; GLUT, glucose transporter; PDE, phosphodiesterase; PKA, protein kinase A. (B) OCI‐AML‐2, OCI‐AML‐3, and KMS11 cells were treated with atorvastatin (4, 2 and 4 µm for OCI‐AML‐2, OCI‐AML‐3, and KMS11 cells, respectively) ± a glucose uptake inhibitor (fasentin; 12.5, 6.3, and 12.5 µm for OCI‐AML‐2, OCI‐AML‐3, and KMS11 cells, respectively), ENT inhibitor (NBMPR; 20 µm), cGMP‐hydrolyzing PDE5 inhibitor (MBMQ; 10 µm), or cAMP‐hydrolyzing PDE3 inhibitor (cilostazol; 25, 12.5, and 25 µm for OCI‐AML‐2, OCI‐AML‐3, and KMS11 cells, respectively). After 48 h, cell viability was evaluated by MTT assays. Data are represented as the mean + SD. *P < 0.05 (one‐way ANOVA with Tukey's multiple comparisons test, where the indicated groups were compared to the other groups of that cell line). ^#^ *P* < 0.05 (one‐way ANOVA with Tukey's multiple comparisons test, comparing the two indicated groups).

Dipyridamole has been reported to have multiple targets and can function as an inhibitor of nucleoside transport [11], glucose uptake [12], and PDEs [13] (Fig. 2A). To test which, if any, of these reported functions of dipyridamole are important for potentiating statin‐induced cancer cell death, we assayed additional compounds with similar activities for their ability to phenocopy dipyridamole. For these experiments, we evaluated the following compounds: NBMPR [equilibrative nucleoside transporter 1 (ENT1) inhibitor], fasentin [glucose transporter 1 (GLUT1) inhibitor], MBMQ (PDE5 inhibitor), and cilostazol (PDE3 inhibitor). AML (OCI‐AML‐2, OCI‐AML‐3) and MM (KMS11) cells were treated with each compound alone or in combination with atorvastatin. The concentrations of each compound were chosen such that they had minimal single‐agent effects on cell viability (< 20%), but were still within the range known to inhibit the target under investigation [14, 15, 16, 17, 18, 19, 20]. Of the four compounds evaluated, only the combination of atorvastatin and cilostazol was observed to decrease AML and MM cell viability in all three cell lines (Fig. 2B). We further demonstrated that these effects were on‐target and not specific to atorvastatin, as a similar decrease in cell viability was observed when cilostazol was combined with fluvastatin, another statin drug (Fig. S1). Moreover, the addition of exogenous MVA or GGPP was able to fully rescue the decrease in cell viability caused by the statin–cilostazol combination (Fig. S1), further supporting that these effects were due to MVA pathway inhibition.

### 3.2 Compounds that increase cAMP levels phenocopy dipyridamole to potentiate statin‐induced apoptosis

PDEs catalyze the hydrolysis of cAMP and cyclic guanosine monophosphate (cGMP) (Fig. 2A), thereby regulating the intracellular concentrations of these secondary messengers. There are 11 PDE proteins that can be expressed in mammalian cells, which differ in their cellular functions, structures, expression patterns, and affinities for cAMP and cGMP [21, 22]. Dipyridamole is known to inhibit multiple cAMP‐ and cGMP‐hydrolyzing PDEs with varying affinities [13, 22]. In contrast, cilostazol is reported to be a specific inhibitor of PDE3, which is a cAMP‐hydrolyzing PDE [13, 21]. Given our observation that the statin–cilostazol combination was uniquely able to decrease the viability of AML and MM cells, we hypothesized that inhibition of cAMP hydrolysis by dipyridamole may be responsible, at least in part, for its ability to synergize with statins to induce cancer cell death. Indeed, dipyridamole treatment, at the concentration used throughout this study (5 μm), resulted in a 2.5‐fold increase in intracellular cAMP levels (Fig. S2).

**Fig. 3:**
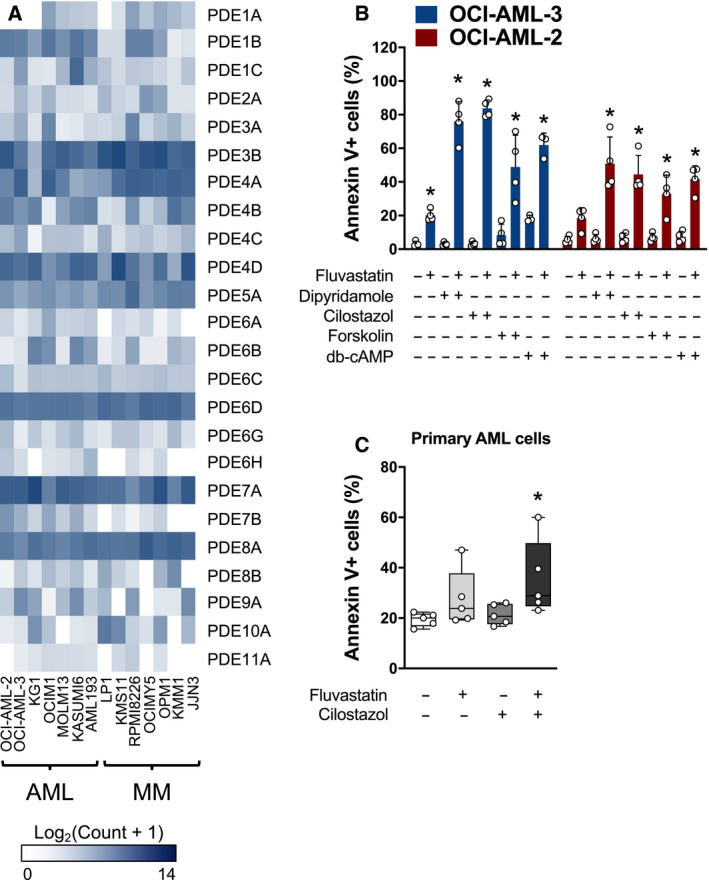
Compounds that increase cAMP levels phenocopy dipyridamole to potentiate statin‐induced apoptosis. (A) RNA expression of the different PDEs in a panel of human AML and MM cell lines. Data were mined from the CCLE database. (B) OCI‐AML‐2 and OCI‐AML‐3 cells were treated with fluvastatin (4 µm for OCI‐AML‐2 and 2 µm for OCI‐AML‐3) ± a PDE3 inhibitor (cilostazol; 20 µm), an adenylate cyclase activator (forskolin; 10 µm) or db‐cAMP (0.1 mm). After 48 h, cells were labeled with FITC‐conjugated Annexin V and apoptotic cells were quantified by flow cytometry. *P < 0.05 (one‐way ANOVA with Dunnett's multiple comparisons test, where the indicated groups were compared to the solvent controls group of that cell line). Data are represented as the mean + SD. (C) Primary AML cells were cultured in the presence of solvent controls, 5 µm fluvastatin, 20 µm cilostazol, or the combination. After 48 h, cells were labeled with FITC‐conjugated Annexin V and analyzed by flow cytometry. Data from four independent AML patient samples are represented as box plots with whiskers depicting the maximum and minimum values. *P < 0.05 (one‐way ANOVA with Dunnett's multiple comparisons test, where the indicated group was compared to the solvent controls group).

To evaluate whether the PDEs targeted by dipyridamole and cilostazol are expressed in AML and MM cells, we mined the Cancer Cell Line Encyclopedia (CCLE) database [8]. Indeed, multiple PDEs, including isoforms of PDE3, PDE5, PDE6, PDE7, and PDE8, are highly and consistently expressed in a panel of AML and MM cell lines, including previously characterized statin‐sensitive (e.g., KMS11, OCI‐AML‐3) and insensitive (e.g., LP1) cell lines (Fig. 3A) [6, 23, 24]. We subsequently evaluated the ability of an adenylate cyclase activator (forskolin) and cell‐permeable analog of cAMP (db‐cAMP) to potentiate statin‐induced apoptosis in AML cells. The combination of fluvastatin and dipyridamole, cilostazol, forskolin, or db‐cAMP significantly induced apoptosis in OCI‐AML‐2 and OCI‐AML‐3 cells, whereas no significant apoptosis was observed in response to treatment with each cAMP‐modulating compound on its own (Fig. 3B). To determine whether primary cells were similarly sensitive to the combination of a statin and PDE inhibitor, we treated primary AML cells with fluvastatin and/or cilostazol for 48 h, after which apoptosis was quantified by Annexin V staining using flow cytometry. Indeed, the fluvastatin–cilostazol combination significantly induced apoptosis in these cells (Fig. 3C). This is consistent with our previous report that the statin–dipyridamole combination can induce apoptosis in primary AML cells [6]. Notably, we evaluated the statin–cilostazol combination in primary cells from three of the same patients as in our previous report with dipyridamole, and observed concordant results [6]. Collectively, these data suggest that elevating intracellular levels of cAMP may be an effective way to sensitize hematological cancer cells to statin‐induced apoptosis.

### 3.3 Compounds that increase cAMP levels differentially modulate sterol metabolism

**Fig. 4:**
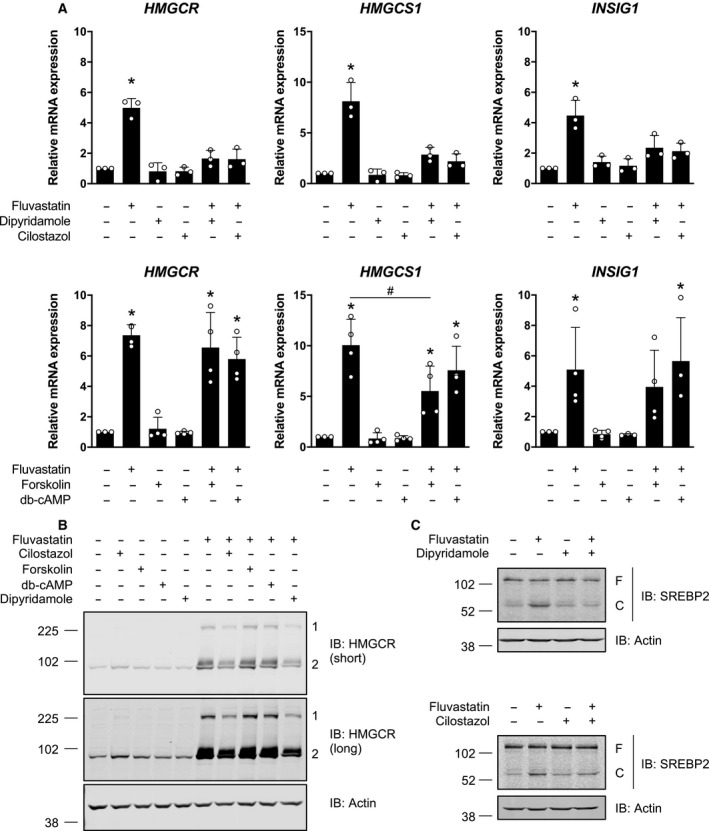
Compounds that increase cAMP levels differentially modulate sterol metabolism. (A) LP1 cells were treated with 4 μm fluvastatin ± 5 μm dipyridamole, 20 μm cilostazol, 10 μm forskolin, or 0.1 mm db‐cAMP for 16 h, and RNA was isolated to assay for *HMGCR*, *HMGCS1* and *INSIG1* expression by qRT–PCR. mRNA expression data are normalized to *GAPDH* expression. Data are represented as the mean + SD. *P < 0.05 (one‐way ANOVA with Tukey's multiple comparisons test, where the indicated groups were compared to the solvent controls group), ^#^ *P* < 0.05 (one‐way ANOVA with Tukey's multiple comparisons test, comparing the two indicated groups). (B) LP1 cells were treated with 4 μm fluvastatin ± 5 μm dipyridamole, 20 μm cilostazol, 10 μm forskolin, or 0.1 mm db‐cAMP for 24 h, and protein was isolated to assay for HMGCR expression by immunoblotting. 1 = HMGCR oligomer, 2 = HMGCR monomer. Immunoblots are representative of three independent experiments. (C) LP1 cells were treated with 4 μm fluvastatin ± 5 μm dipyridamole or 20 μm cilostazol for 8 h, and protein was isolated to assay for SREBP2 cleavage (activation) by immunoblotting. F, full‐length SREBP2; C, cleaved SREBP2. Immunoblots are representative of three independent experiments.

We previously demonstrated that dipyridamole inhibits statin‐induced SREBP2 cleavage and activation, which sensitizes cancer cells to statin‐induced apoptosis [4, 6]. To test whether compounds that increase cAMP levels similarly inhibit the induction of sterol metabolism gene expression in response to statin treatment, we treated LP1 cells with fluvastatin as a single agent or in combination with a PDE inhibitor (dipyridamole or cilostazol), forskolin or db‐cAMP, and then evaluated the expression of three SREBP2 target genes by qRT–PCR: *HMGCR*, HMG‐CoA synthase 1 (*HMGCS1*), and insulin‐induced gene 1 (*INSIG1*). We chose LP1 cells for these experiments because we previously demonstrated that this cell line robustly activates SREBP2 in response to statin exposure, and cotreatment with dipyridamole sensitizes them to statin‐induced apoptosis [6]. As expected, treatment of LP1 cells with fluvastatin resulted in the induction of all three sterol‐regulated genes, a response which was completely blocked by dipyridamole cotreatment (Fig. 4A). Cilostazol similarly inhibited fluvastatin‐induced expression of these SREBP2 target genes (Fig. 4A). In contrast, forskolin and db‐cAMP had weaker, if any, effects on the expression of these sterol‐regulated genes in this cell line, and yet both compounds potentiated statin‐induced apoptosis (Figs 3B and 4A, Fig. S3). Concordantly, only dipyridamole and cilostazol decreased statin‐induced HMGCR protein expression (Fig. 4B), which was associated with the inhibition of SREBP2 cleavage following statin treatment (Fig. 4C).

cAMP can regulate several effectors, the most well studied of which is cAMP‐dependent protein kinase A (PKA). PKA phosphorylates a multitude of proteins with diverse roles in signal transduction, metabolism, ion transport, and transcription regulation [25]. In particular, PKA has been shown to phosphorylate and negatively regulate SREBP1 (the master transcriptional regulator of fatty acid biosynthesis) *in vitro* at a residue that is conserved between SREBP1 and SREBP2 [26]. However, given our observation that db‐cAMP did not inhibit statin‐induced SREBP2 target gene expression (Fig. 4A), we reasoned that the effects of dipyridamole and cilostazol on SREBP2 were likely independent of cAMP/PKA signaling. To validate this model, we knocked out the alpha catalytic subunit of PKA (PKA Cα, encoded by *PRKACA*) in LP1 cells and evaluated the subsequent effects on dipyridamole and cilostazol activity. Consistent with a cAMP/PKA‐independent mechanism, both dipyridamole and cilostazol retained their ability to inhibit SREBP2 and potentiate statin‐induced cell death in PKA‐depleted LP1 cells (Figs S4 and S5).

To further confirm the above observation, we evaluated dipyridamole and cilostazol activity in isogenic wild‐type and PKA‐null (kin‐) S49 cells [27]. S49 kin‐ cells have no detectable PKA activity due to improper *cis*‐autophosphorylation at serine 338 during translation, which renders the catalytic subunit of PKA insoluble [28]. Indeed, dipyridamole and cilostazol potentiated statin‐induced cell death in both S49 wild‐type and kin‐ cells (Fig. S4).

**Fig. 5:**
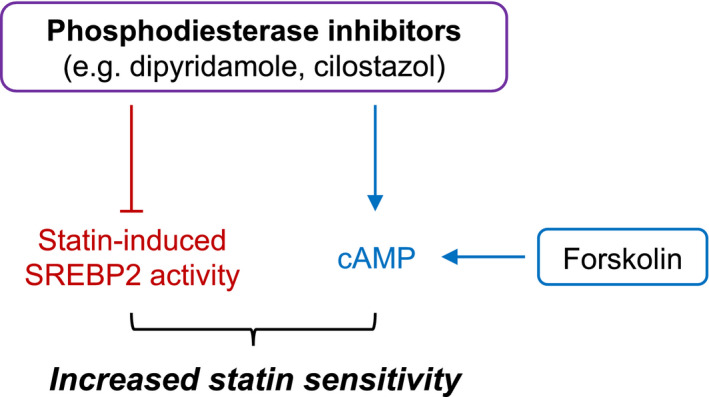
Proposed model for how cAMP‐hydrolyzing PDE inhibitors potentiate statin‐induced cancer cell death. Compounds that increase intracellular cAMP levels, including PDE inhibitors (e.g., dipyridamole, cilostazol) and forskolin, can sensitize cancer cells to statin‐induced apoptosis. Dipyridamole and cilostazol also inhibit statin‐induced activation of SREBP2 through a cAMP‐independent mechanism, which abrogates the restorative feedback loop of the MVA pathway and further sensitizes cancer cells to statin‐induced apoptosis.

Taken together, these data suggest that compounds that increase cAMP levels, including PDE inhibitors and forskolin, can sensitize hematological cancer cells to statin‐induced apoptosis. Furthermore, PDE inhibitors such as dipyridamole and cilostazol further possess cAMP/PKA‐independent activity against statin‐induced SREBP2 activation (Fig. 5).

## 4 Discussion

Our laboratory previously reported a novel role for the drug dipyridamole as an inhibitor of the SREBP family of transcription factors [4, 6]. As a result, dipyridamole can sensitize certain cancer cells to statin‐induced apoptosis (Fig. 1) [4, 6]. However, given the polypharmacology of dipyridamole, the mechanism by which it inhibits the SREBP proteins and synergizes with statins remains to be fully understood. As a step toward elucidating this mechanism, we evaluated individual compounds that phenocopied the different known functions of dipyridamole for their ability to sensitize AML and MM cell lines to statin‐induced cell death. Through this approach, we were able to dissect the polypharmacology of dipyridamole and implicate its role as a cAMP‐hydrolyzing PDE inhibitor in potentiating statin‐induced apoptosis.

Our study revealed that cAMP‐hydrolyzing PDE inhibitors, including dipyridamole and cilostazol, sensitize hematological cancer cells to statin‐induced apoptosis via a dual mechanism (Fig. 5). By inhibiting PDE activity, dipyridamole and cilostazol increase intracellular cAMP levels (Fig. S2) [18]. We demonstrated that other compounds that increase cAMP levels, including forskolin, similarly sensitize cancer cells to statin‐induced cell death. Importantly, cotreatment with a statin and cAMP‐modulating agent was effective at potentiating cell death in both statin‐sensitive (e.g., KMS11, OCI‐AML‐3) and statin‐insensitive (e.g., LP1) cell lines (Fig. 3B, Figs S1, S3,and S4D). Our data are consistent with a previous report, where the combination of lovastatin and db‐cAMP was shown to enhance differentiation and cytotoxicity in embryonal carcinoma and neuroblastoma cell lines [29]. However, the critical cAMP‐regulated effector that modulates statin sensitivity in cancer cells remains to be identified. In the present study, we found that dipyridamole and cilostazol potentiate statin‐induced cell death in a PKA‐independent manner (Fig. S4). In addition to PKA, cAMP also regulates specific ion channels and the EPAC (exchange protein directly activated by cAMP) proteins, which are cAMP‐dependent guanine nucleotide exchange factors for the RAP GTPases [30]. Future work is required to delineate the mechanism by which elevated cAMP levels sensitize cancer cells to statin‐induced apoptosis.

We further demonstrated that the PDE inhibitors dipyridamole and cilostazol inhibit the SREBP2‐regulated feedback mechanism of the MVA pathway via an additional, cAMP‐independent mechanism (Fig. 4). Interestingly, cilostazol has previously been reported to inhibit insulin‐induced expression of SREBP1 [31], but the potential involvement of cAMP signaling was not explored. Data in the literature are conflicting as to the effects of PDE inhibitors on lipid metabolism. A recent study demonstrated that combined inhibition of PDE4 and PDE8 in Leydig cells promotes SREBP2 signaling, cholesterol metabolism, and steroidogenesis [32]. In contrast, data from a randomized controlled trial in patients with type 2 diabetes revealed that cilostazol treatment significantly lowered serum triglyceride and low‐density lipoprotein (LDL) cholesterol levels [33]. The data we present here clearly show that dipyridamole (a pan‐PDE inhibitor) and cilostazol (a PDE3 inhibitor) can abrogate SREBP2 cleavage and activation in AML and MM cells exposed to a statin. It is therefore possible that different PDEs play unique roles in regulating SREBP2 signaling and sterol metabolism and that PDE‐mediated regulation of SREBP2 is tissue type‐ and context‐dependent. In the context of cancer, dipyridamole has been shown to inhibit statin‐induced SREBP2 cleavage and activation in AML, MM, breast cancer, and prostate cancer cells [3, 4, 6], suggesting similar regulation in many different cell types. A rigorous analysis of the effects of different PDE inhibitors on lipid metabolism and investigation into the mechanism(s) by which these clinically approved drugs act to modulate cancer cell metabolism should be a focus of future studies. Interestingly, unlike many other PDE inhibitors, dipyridamole and cilostazol also inhibit adenosine uptake [11, 34]. While we did not observe enhanced cell death when the adenosine reuptake inhibitor NBMPR was combined with a statin (Fig. 2B), it remains possible that dipyridamole and cilostazol inhibit sterol metabolism via a PDE‐independent mechanism or through simultaneous modulation of multiple targets.

The data presented here may have important clinical implications, as many cAMP‐hydrolyzing PDE inhibitors are approved for several nononcology indications [21]. For example, cilostazol (marketed as Pletal) is currently approved and widely used to treat intermittent claudication. The overexpression of several PDEs has been observed in solid and hematological tumors, and the possibility of cAMP‐hydrolyzing PDE inhibition as an anticancer strategy has been preclinically explored alone or in combination with chemo‐ and targeted molecular therapies [35, 36, 37, 38, 39, 40]. In hematological malignancies, primary chronic lymphocytic leukemia patient samples were found to have PDE7B overexpression and noted to be sensitive to PDE7 inhibition in a cAMP‐dependent manner [38]. Another study found a strong synergistic combinatorial effect between adenosine A2A receptor agonists and cAMP‐hydrolyzing PDE inhibitors in MM and diffuse large B‐cell lymphoma cell lines and primary patient samples [41]. Given that a number of PDE inhibitors are poised for repurposing and that statins have demonstrated anticancer activity in early‐phase clinical trials [42, 43, 44, 45, 46, 47, 48, 49], further studies are needed to evaluate the therapeutic benefit of a statin‐PDE inhibitor combination for the treatment of cancer. As the combination of cilostazol and statins has already been evaluated clinically in healthy subjects [50, 51] and in patients with cardiovascular indications [52, 53] without added adverse effects, there is the possibility of fast‐tracking these agents to phase II trials in AML and MM.

## 5 Conclusion

In summary, we propose a working model whereby cAMP‐hydrolyzing PDE inhibitors, such as dipyridamole and cilostazol, increase cAMP levels and inhibit SREBP2 activation via independent mechanisms, both of which converge to potentiate statin‐induced apoptosis in hematological cancer cells (Fig. 5). Given that statins and a number of PDE inhibitors are already approved for various nononcology indications, future studies are needed to thoroughly evaluate the potential therapeutic benefit of these agents for the treatment of hematological malignancies. Moreover, our experimental approach to dissect the polypharmacology of dipyridamole is one that may be useful when interrogating novel functions of other repurposed drugs.

## Conflict of interest

The authors declare no conflict of interest.

## Author contributions

JL, AAP, and LZP conceived and designed the study. JL, AAP, and PS performed experiments, as well as analyzed and interpreted the experimental data. MDM and ADS provided the primary AML cells and clinical expertise. JL, AAP, and LZP wrote the manuscript. All authors read and approved the manuscript. LZP supervised the study.

## Supporting information


**Fig. S1.** Statin‐cilostazol‐induced cancer cell death can be rescued by exogenous MVA or GGPP. KMS11 and OCI‐AML‐3 cells were treated as indicated with fluvastatin (2 µM for KMS11 and 0.5 µM for OCI‐AML‐3 cells), cilostazol (12.5 µM), mevalonate (0.2 mM) and/or GGPP (2 µM). After 48 hr, cell viability was evaluated by MTT assays. Data are represented as the mean + SD. *p < 0.05 (one‐way ANOVA with Tukey's multiple comparisons test, where the indicated groups were compared to the other groups of that cell line).

**Fig. S2.** Dipyridamole treatment increases intracellular cAMP. OCI‐AML‐3 cells were treated with 2 μM fluvastatin ± 5 μM dipyridamole for 15 min and intracellular cAMP levels were quantified. Data are represented as the mean + SD. *p < 0.05 (one‐way ANOVA with Dunnett's multiple comparisons test, where the indicated groups were compared to the solvent controls group).

**Fig. S3.** Forskolin and db‐cAMP sensitize LP1 cells to fluvastatin‐induced apoptosis. LP1 cells were treated with 4 μM fluvastatin ± 10 μM forskolin or 0.1 mM db‐cAMP for 48 hr, after which apoptotic cells (double Annexin V‐positive and 7AAD‐positive cells) were quantified by flow cytometry. Data are represented as the mean + SD. *p < 0.05 (one‐way ANOVA with Dunnett's multiple comparisons test, where the indicated groups were compared to the solvent controls group).

**Fig. S4.** Potentiation of statin‐induced cancer cell death by dipyridamole and cilostazol is independent of PKA. (A) Immunoblot for PKA C‐α expression in LP1 cells expressing Cas9 and a sgRNA to a random locus on chromosome 10 (gC10 Random) or one of two different locations within *PRKACA* (representative of three independent experiments). (B) LP1 gC10 Random and gPRKACA sublines were treated with a range of fluvastatin concentrations (0‐24 µM) ± 5 µM dipyridamole or 10 µM cilostazol. After 48 hr, cell viability was evaluated by MTT assays. The area under each fluvastatin dose‐response curve is plotted. Data are represented as the mean + SD. *p < 0.05 (one‐way ANOVA with Dunnett's multiple comparisons test, where the indicated groups were compared to the fluvastatin alone group of that subline). (C) Immunoblot for PKA C‐α expression in S49 wildtype (WT) or kin‐ (PKA‐null) cells (representative of three independent experiments). (D) S49 WT and kin‐ cells were treated with 5 μM fluvastatin ± 2.5 μM dipyridamole or 5 μM cilostazol for 48 hr, fixed in ethanol and assayed for DNA fragmentation (% pre‐G1 population) as a marker of cell death by propidium iodide staining. Data are represented as the mean + SD. *p < 0.05 (one‐way ANOVA with Dunnett's multiple comparisons test, where the indicated groups were compared to the solvent controls group of that cell line).

**Fig. S5.** Dipyridamole and cilostazol inhibit the sterol‐regulated feedback loop of the MVA pathway independent of PKA. (A) LP1 gPRKACA sublines were treated with 4 μM fluvastatin ± 5 μM dipyridamole or 20 μM cilostazol for 16 hr, and RNA was isolated to assay for *HMGCS1* expression by qRT‐PCR. mRNA expression data are normalized to *GAPDH* expression. Data are represented as the mean + SD. *p < 0.05 (one‐way ANOVA with Sidak's multiple comparisons test, where the indicated groups were compared to the solvent controls group of that subline). (B) LP1 gC10 Random or gPRKACA #1 cells were treated with 4 μM fluvastatin ± 5 μM dipyridamole or 20 μM cilostazol for 8 hr, and protein was isolated to assay for SREBP2 cleavage (activation) by immunoblotting. F = full‐length SREBP2, C = cleaved SREBP2. Immunoblots are representative of three independent experiments.

## Data Availability

The RNA expression data in Fig. 3A were obtained through the CCLE database [8]. All other raw data are available from the corresponding author upon reasonable request.
