# Supplementary figures and images for: Cyclic AMP‐hydrolyzing phosphodiesterase inhibitors potentiate statin‐induced cancer cell death
Source: Mol Oncol. 2020 Aug 25;14(10):2533–45. doi: 10.1002/1878-0261.12775 (PMC7530792; doi:10.1002/1878-0261.12775)

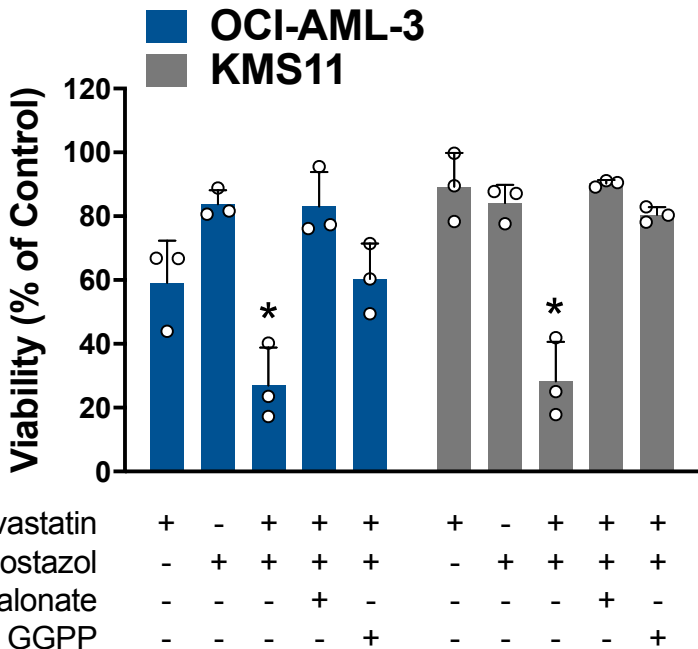

Supplement: Supplementary file 1 — Fig. S1. Statin‐cilostazol‐induced cancer cell death can be rescued by exogenous MVA or GGPP. KMS11 and OCI‐AML‐3 cells were treated as indicated with fluvastatin (2 µM for KMS11 and 0.5 µM for OCI‐AML‐3 cells), cilostazol (12.5 µM), mevalonate (0.2 mM) and/or GGPP (2 µM). After 48 hr, cell viability was evaluated by MTT assays. Data are represented as the mean + SD. *p < 0.05 (one‐way ANOVA with Tukey's multiple comparisons test, where the indicated groups were compared to the other groups of that cell line). [file MOL2-14-2533-s001.pdf]

## OCI-AML-3

Intracellular cAMP  
(Fold change)

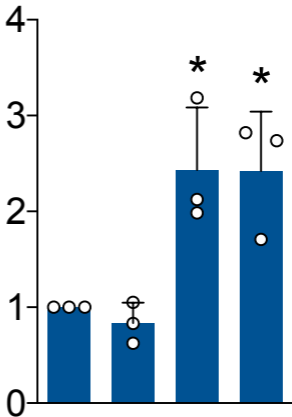

Fluvastatin  
Dipyridamole

|   |   |   |   |
|---|---|---|---|
| - | + | - | + |
| - | - | + | + |

Supplement: Supplementary file 2 — Fig. S2. Dipyridamole treatment increases intracellular cAMP. OCI‐AML‐3 cells were treated with 2 μM fluvastatin ± 5 μM dipyridamole for 15 min and intracellular cAMP levels were quantified. Data are represented as the mean + SD. *p < 0.05 (one‐way ANOVA with Dunnett's multiple comparisons test, where the indicated groups were compared to the solvent controls group). [file MOL2-14-2533-s002.pdf]

# LP1

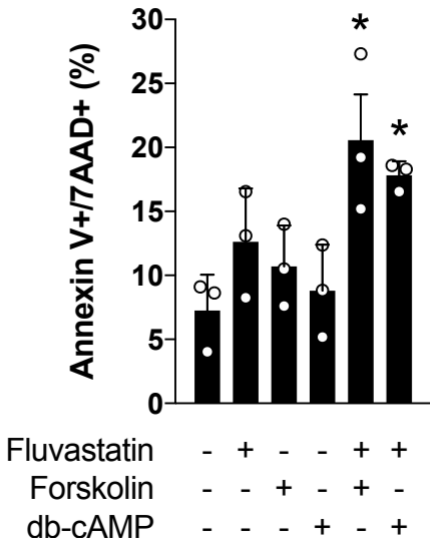

Supplement: Supplementary file 3 — Fig. S3. Forskolin and db‐cAMP sensitize LP1 cells to fluvastatin‐induced apoptosis. LP1 cells were treated with 4 μM fluvastatin ± 10 μM forskolin or 0.1 mM db‐cAMP for 48 hr, after which apoptotic cells (double Annexin V‐positive and 7AAD‐positive cells) were quantified by flow cytometry. Data are represented as the mean + SD. *p < 0.05 (one‐way ANOVA with Dunnett's multiple comparisons test, where the indicated groups were compared to the solvent controls group). [file MOL2-14-2533-s003.pdf]

**A**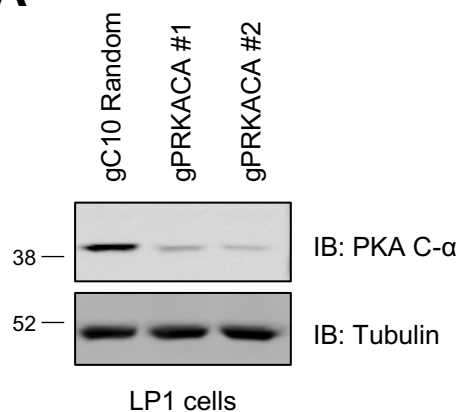**B**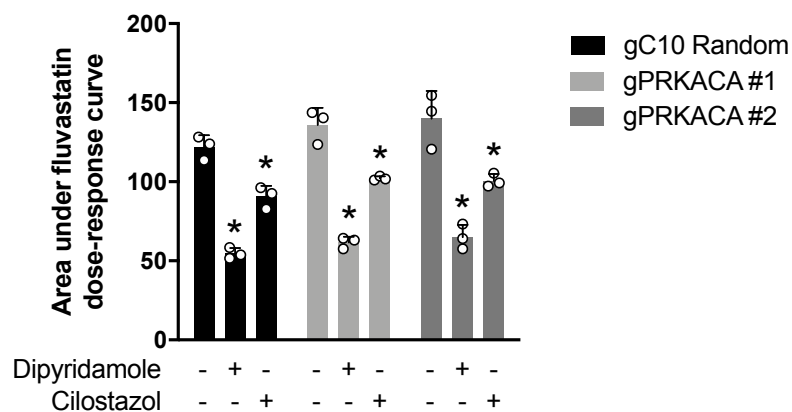**C**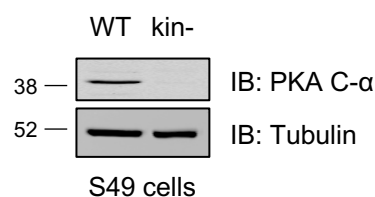**D**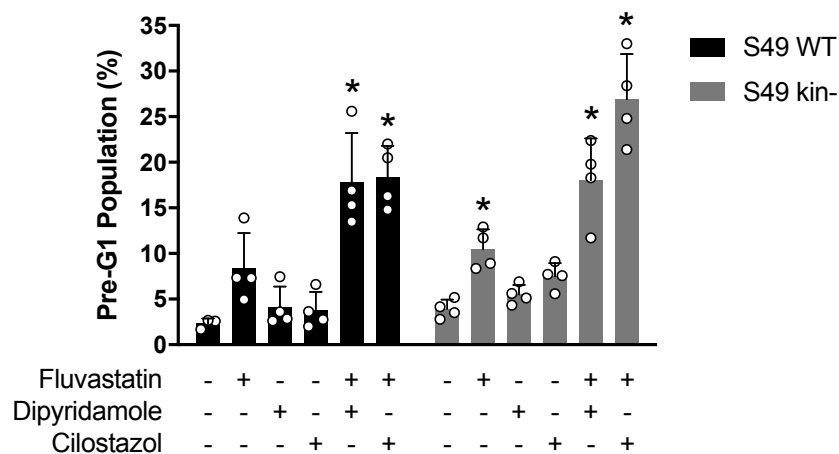

Supplement: Supplementary file 4 — Fig. S4. Potentiation of statin‐induced cancer cell death by dipyridamole and cilostazol is independent of PKA. (A) Immunoblot for PKA C‐α expression in LP1 cells expressing Cas9 and a sgRNA to a random locus on chromosome 10 (gC10 Random) or one of two different locations within PRKACA (representative of three independent experiments). (B) LP1 gC10 Random and gPRKACA sublines were treated with a range of fluvastatin concentrations (0‐24 µM) ± 5 µM dipyridamole or 10 µM cilostazol. After 48 hr, cell viability was evaluated by MTT assays. The area under each fluvastatin dose‐response curve is plotted. Data are represented as the mean + SD. *p < 0.05 (one‐way ANOVA with Dunnett's multiple comparisons test, where the indicated groups were compared to the fluvastatin alone group of that subline). (C) Immunoblot for PKA C‐α expression in S49 wildtype (WT) or kin‐ (PKA‐null) cells (representative of three independent experiments). (D) S49 WT and kin‐ cells were treated with 5 μM fluvastatin ± 2.5 μM dipyridamole or 5 μM cilostazol for 48 hr, fixed in ethanol and assayed for DNA fragmentation (% pre‐G1 population) as a marker of cell death by propidium iodide staining. Data are represented as the mean + SD. *p < 0.05 (one‐way ANOVA with Dunnett's multiple comparisons test, where the indicated groups were compared to the solvent controls group of that cell line). [file MOL2-14-2533-s004.pdf]

**A*****HMGCS1***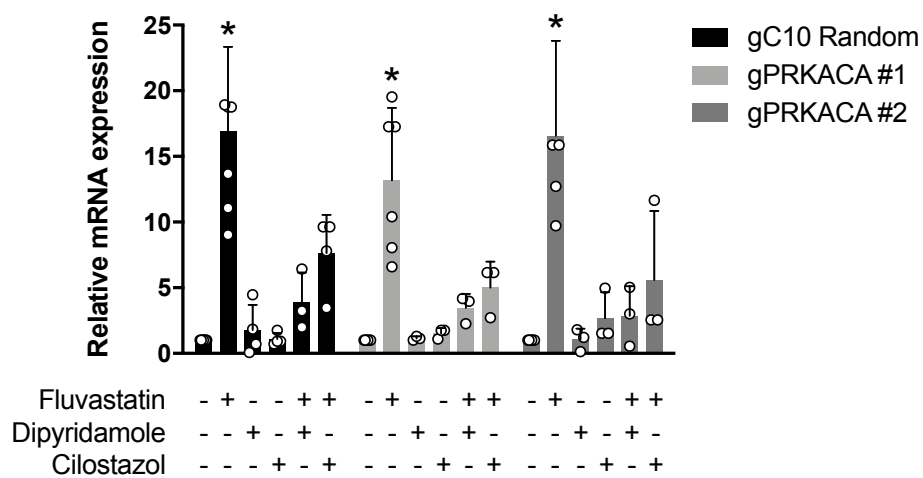**B**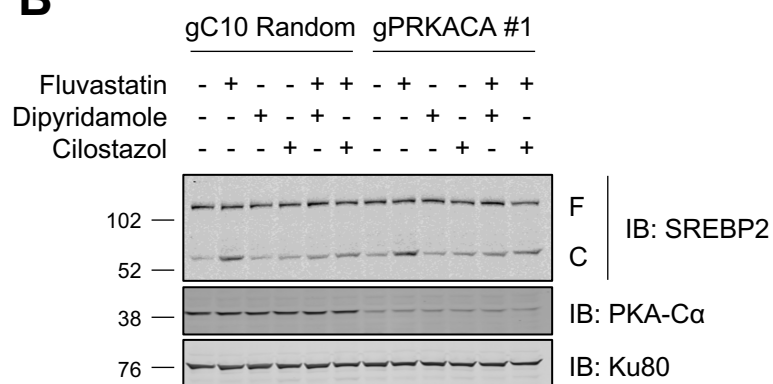

Supplement: Supplementary file 5 — Fig. S5. Dipyridamole and cilostazol inhibit the sterol‐regulated feedback loop of the MVA pathway independent of PKA. (A) LP1 gPRKACA sublines were treated with 4 μM fluvastatin ± 5 μM dipyridamole or 20 μM cilostazol for 16 hr, and RNA was isolated to assay for HMGCS1 expression by qRT‐PCR. mRNA expression data are normalized to GAPDH expression. Data are represented as the mean + SD. *p < 0.05 (one‐way ANOVA with Sidak's multiple comparisons test, where the indicated groups were compared to the solvent controls group of that subline). (B) LP1 gC10 Random or gPRKACA #1 cells were treated with 4 μM fluvastatin ± 5 μM dipyridamole or 20 μM cilostazol for 8 hr, and protein was isolated to assay for SREBP2 cleavage (activation) by immunoblotting. F = full‐length SREBP2, C = cleaved SREBP2. Immunoblots are representative of three independent experiments. [file MOL2-14-2533-s005.pdf]
